# Advancing Geriatric Care: Harnessing AI and Human Expertise to Enhance CNA Training

**DOI:** 10.1093/geroni/igaf122.3511

**Published:** 2025-12-31

**Authors:** Suzanne White, Stephanie Trotter, Robin McAtee, Valerie Bearden

**Affiliations:** College of Nursing - University of Arkansas for Medical Sciences, Little Rock, Arkansas, United States; University of Arkansas for Medical Sciences, Little Rock, Arkansas, United States; University of Arkansas for Medical Sciences, Little Rock, Arkansas, United States; Oaklawn Center on Aging, Hot Springs, Arkansas, United States

## Abstract

Staff of the Arkansas Geriatric Education Collaborative (AGEC), a HRSA-funded Geriatric Workforce Enhancement Program, employed a strategic collaboration between human expertise and artificial intelligence (AI), specifically ChatGPT, a Large Language Model (LLM) to develop a robust 40-hour Advanced Geriatric Specialty curriculum for Certified Nursing Assistants (CNAs). Subject matter experts (SMEs) first conducted a thorough review of the Arkansas State CNA curriculum, identifying opportunities to supplement geriatric knowledge and practice. Based on this analysis, they developed a list of learning module topics to include in the specialty curriculum, beginning with an introduction to the 4Ms of Age-Friendly Care – What Matters, Medication, Mentation, and Mobility. ChatGPT was then employed to synthesize information from reputable sources for each topic and accelerate the generation of content for each module, linking content across modules to the 4Ms where relevant. Each module included a scenario-based application activity or a reflection exercise to reinforce learning and encourage critical thinking. Pre/post tests were also generated to assess the learning of key concepts. Subject matter experts reviewed and refined all AI-generated content to ensure accuracy, clinical relevance, and alignment with best practices in geriatric care. This innovative integration of AI-driven content development with expert oversight allowed for the accelerated creation of a high-quality, evidence-informed curriculum to enhance geriatric competency for CNAs.

